# Testosterone or Estradiol When Implanted in the Medial Preoptic Nucleus Trigger Short Low-Amplitude Songs in Female Canaries

**DOI:** 10.1523/ENEURO.0502-18.2019

**Published:** 2019-05-07

**Authors:** Laura M. Vandries, Samar Ghorbanpoor, Gilles Cornez, Olesya T. Shevchouk, Gregory F. Ball, Charlotte A. Cornil, Jacques Balthazart

**Affiliations:** 1 GIGA Neurosciences, University of Liege, 4000 Liège, Belgium; 2 Department of Psychology, University of Maryland, College Park, MD 20742

**Keywords:** medial preoptic nucleus, preoptic area, singing motivation, song control system, songbirds, testosterone

## Abstract

In male songbirds, the motivation to sing is largely regulated by testosterone (T) action in the medial preoptic area, whereas T acts on song control nuclei to modulate aspects of song quality. Stereotaxic implantation of T in the medial preoptic nucleus (POM) of castrated male canaries activates a high rate of singing activity, albeit with a longer latency than after systemic T treatment. Systemic T also increases the occurrence of male-like song in female canaries. We hypothesized that this effect is also mediated by T action in the POM. Females were stereotaxically implanted with either T or with 17β-estradiol (E2) targeted at the POM and their singing activity was recorded daily during 2 h for 28 d until brains were collected for histological analyses. Following identification of implant localizations, three groups of subjects were constituted that had either T or E2 implanted in the POM or had an implant that had missed the POM (Out). T and E2 in POM significantly increased the number of songs produced and the percentage of time spent singing as compared with the Out group. The songs produced were in general of a short duration and of poor quality. This effect was not associated with an increase in HVC volume as observed in males, but T in POM enhanced neurogenesis in HVC, as reflected by an increased density of doublecortin-immunoreactive (DCX-ir) multipolar neurons. These data indicate that, in female canaries, T acting in the POM plays a significant role in hormone-induced increases in the motivation to sing.

## Visual Abstract

**Figure:**
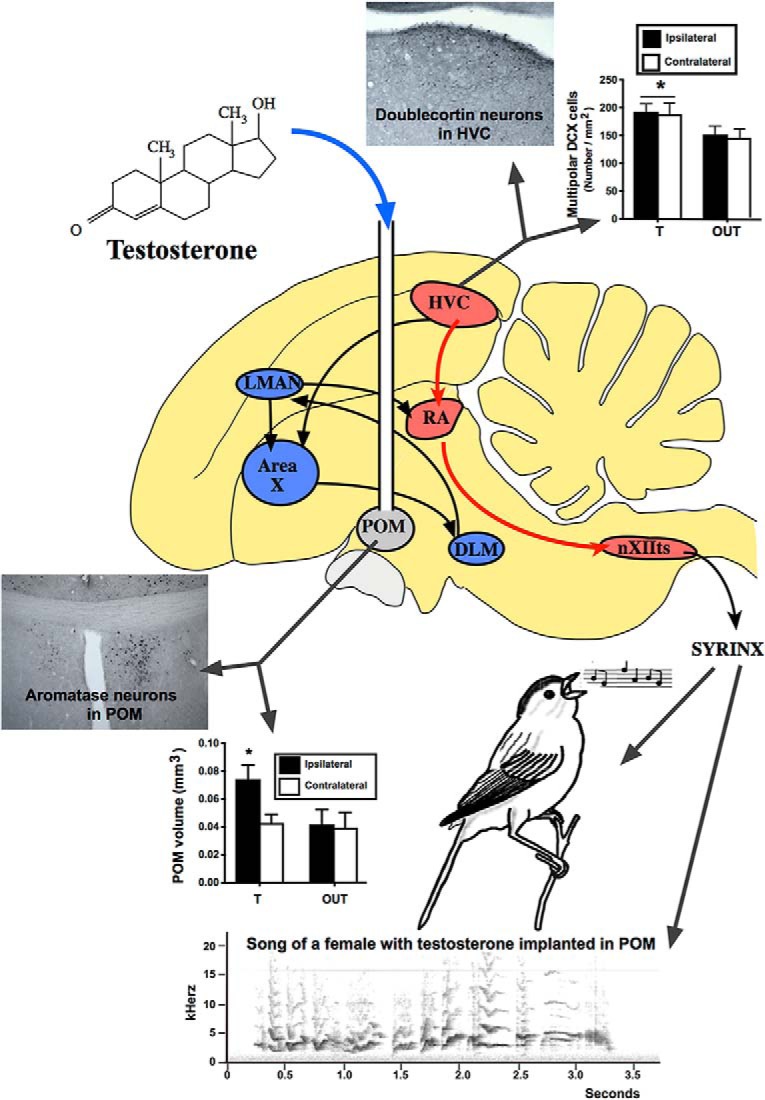

## Significance Statement

Systemic testosterone (T) increases male-like song in adult female canaries. We demonstrate by stereotaxic implantation of T or 17β-estradiol (E2) that this effect is mediated, as has been demonstrated in males, by hormone action in the preoptic area. These implants significantly increased the number of songs produced and the percentage of time spent singing, but the songs produced remained short in duration and simple in structure. This singing activity did not result in an increase in HVC volume, as observed in males, but there was an enhanced density of doublecortin-immunoreactive (DCX-ir) new neurons supporting the notion that HVC neurogenesis is at least in part activity–dependent. These data also indicate that neural mechanisms regulating T-induced singing are similar in males and females.

## Introduction

Male song produced by songbirds (members of the suborder Passeres or Oscines) functions to promote territory defense and to attract female mates (Collins, 2004; Catchpole and Slater, 2008). Male song is therefore often produced, especially among species in the temperate zone, at its highest rates and in its most stereotypic fashion during the breeding season (Catchpole and Slater, 2008; Schlinger and Brenowitz, 2017). Both the high rate of singing and the high degree of stereotypy are facilitated by testosterone (T) acting in males at brain targets via androgenic and estrogenic metabolites (Harding, 2008; Schlinger and Brenowitz, 2017).

In male canaries specifically, there is clear evidence that song rate and quality and the morphology of the song system are regulated by seasonal changes in T (Nottebohm et al., 1986, 1987). Androgenic and estrogenic metabolites of T seem to be involved in these processes (Fusani et al., 2003; Fusani and Gahr, 2006). The effects of T on these different components of song production are mediated by T acting in distinct areas of the brain (Alward et al., 2017b). T in the preoptic area is important for effects on song rate (Alward et al., 2013), while T acting on nuclei in the song control system, such as HVC or the robust nucleus of the arcopallium (RA), is important for effects on song stereotypy (Alward et al., 2016, 2017a).

Female songbirds also sing in some species and, although there is evidence that female song is actually an ancestral feature in the passerine order (Odom et al., 2014), much less is known about the function and neuroendocrine control of female song (Odom and Benedict, 2018). The specialized neural circuit regulating song tends to contain brain nuclei of larger volume in males than in females, even in species where females sing at a higher or similar rate than males (Gahr et al., 2008; Ball, 2016). However, there is a rough relationship between brain variation and sex differences in behavior in that the sex difference in song nuclei volumes tends to be more robust in species with little or no female song as compared to species where females produce substantial song (MacDougall-Shackleton and Ball, 1999; Ball et al., 2008). The role of hormones in adult song production in females and where they might act is, however, not well understood.

Female canaries only sing very infrequently very short primitive songs and correlatively the volume of their song control nuclei is two to five times smaller than in males (Nottebohm and Arnold, 1976). Interestingly, treating adult female canaries with male-typical concentrations of T does increase the volume of their song control nuclei and makes their song more male-like in rate and complexity (Nottebohm, 1980; Hartog et al., 2009), although this sex difference in brain and behavior cannot be completely reversed based on adult hormone treatment (Madison et al., 2015).

The ability of exogenous T to stimulate more male-like song in adult female canaries provides an opportunity to study where and how hormones can act in the female brain to regulate song production, a male-typical behavior. Specifically, we employed here stereotaxic procedures to ask whether T or its estrogenic metabolite, 17β-estradiol (E2), act in the preoptic area of female canaries to regulate song rate. These females were compared to females which also had received a T or E2 brain implant, but in which the implant had missed its intended target (the preoptic area) and was therefore presumably unable to activate singing behavior. We show that in females the medial preoptic area plays a key role in the control of the singing motivation as has been shown in males. This study also demonstrates that activating singing results in an increased neurogenesis in the telencephalic song control area HVC, which brings additional support to the idea that this neurogenesis is at least in part activity dependent.

## Materials and Methods

### Subjects and experimental procedures

This experiment was performed on a total of 32 adult female canaries (*Serinus canaria*) of the Fife fancy breed that were obtained as adults from a breeding colony established at the University of Antwerp, Belgium. Birds were kept on a short-day photoperiod (8/16 h light/dark cycle) between their arrival in the laboratory and the beginning of the experiment. At that time females were isolated in one of our 16 custom-built sound-attenuated boxes and their vocal behavior was recorded for 2 h in the morning for 2 d to ensure that they were not singing.

Sound was acquired from all 16 channels simultaneously via custom-made microphones (microphone from Projects Unlimited/Audio Products Division, amplifier from Maxim Integrated) and an Allen & Heath ICE-16 multichannel recorder. The sound file was acquired and saved as a .wav file by Raven v1.4 software (Bioacoustics Research Program 2011; Raven Pro: Interactive Sound Analysis Software, version 1.4, The Cornell Lab of Ornithology) at a sampling frequency of 44,100 Hertz.

During the next 2 d each female received a stereotaxic implant of T or E2 aimed at the medial preoptic nucleus (POM). Brain implants were prepared, filled with crystalline T or crystalline E2 and implanted into the POM following a previously published procedure (Alward et al., 2013). Briefly, implants were prepared using blunted 27-gauge needles filled over a length of 1 mm with crystalline T or E2. Under isoflurane anesthesia subjects were fixed in a stereotaxic apparatus with ear bars and a beak holder holding the head in a standardized position. The following stereotaxic coordinates were used to target the POM: dorsoventral: −6.5 mm from the dorsal surface of the brain; anterior-posterior: 2.2 mm from the rostral tip of the cerebellum; and medio-lateral: ±0.15 mm from midline. Half of the subjects in each group were implanted on the left side of the brain and half on the right side.

The skull immediately over these coordinates was removed with a micro-drill, the implant was lowered to the targeted position, dental cement was applied around the implant and the skin was sutured. The bird was placed under a heat lamp to recover until perching. Birds were returned to their sound-attenuated box where photoperiod was switched to 16/8 h light/dark to photostimulate the birds mimicking a reproductive state (Hurley et al., 2008) and their vocalizations were then recorded for 30 d during 2 h daily immediately following lights-on (9 A.M.).

There were 16 recording boxes available for this experiment which was therefore run in two successive cohorts with the exact same procedure. In the first cohort all 16 females were implanted with T to test specifically the effect of this steroid, but one died soon thereafter. Since positive results had been obtained with T, the second cohort was mostly used to test the effects of E2. Twelve females were thus implanted with E2, but four females received T to provide an internal control between cohorts. We did not treat additional subjects with empty implants because it was anticipated that in a substantial number of birds the implant targeted to the POM would actually miss its target, so that these subjects could be used as negative controls. Previous work in canaries indeed showed that empty implants and T-filled implants that miss their target produce similar behavioral results (Alward et al., 2013, 2016).

All experimental procedures complied with Belgian laws concerning the Protection and Welfare of Animals and the Protection of Experimental Animals, and experimental protocols were approved by the Ethics Committee for the Use of Animals of the University of Liege (protocol number 1739). In all housing situations, food, water, baths, cuttlebone, and grit were available *ad libitum*.

### Brain collection and sectioning

Canaries were deeply anesthetized with 0.04 ml of Nembutal. Once reflexes had stopped, birds were perfused through the heart with phosphate-buffered saline (PBS, 1.43 g/l Na_2_HPO_4_, 0.48 g/l KH_2_PO4, 7.2 g/l NaCl) until return flow in the atrium was clear, followed by 4% paraformaldehyde (Sigma) in PBS 0.1 M. Brains were dissected out of the skull and post-fixed overnight in the same fixative solution. The syrinx and ovary were extracted and weighed, and the cloacal protuberance (length × width) was measured. On the next day, brains were rinsed in PBS and transferred to 30% sucrose in PB 0.1M stored at 4°C until they sank. They were then frozen on dry ice and stored at –80°C until used.

Brains were notched on the left side, then cut into four series of 30-μm-thick coronal sections with a Leica CM 3050S cryostat. The sections were collected in Tris buffered-saline (TBS; 0.05 M Tris and 0.9% NaCl; pH 7.6). Sections were stored in a cryoprotective solution (0.01 M PBS with 10 g/l polyvinylpyrrolidone, 300 g/l sucrose, and 300 ml/l ethylene glycol) and stored at –20°C until used.

### Nissl staining

The first series of sections was mounted on Superfrost slides and left to dry overnight. After rehydration in baths of decreasing concentrations of isopropanol, slides were stained with toluidine blue and differentiated in Walpole buffer and molybdate buffer. The sections were then dehydrated in increasing concentrations of isopropanol and lastly in xylene and coverslipped with Eukitt mounting medium (Sigma). These sections were later used to identify the implants location and to determine HVC volumes.

### DCX immunohistochemistry

The second series of brain sections was stained by immunohistochemistry for doublecortin (DCX), a marker of young new neurons in the canary HVC (Balthazart and Ball, 2014; Balthazart et al., 2008), to quantify neurogenesis in HVC and its periphery and obtain a second independent measure of HVC volume by techniques previously described and validated for canaries (Boseret et al., 2007; Balthazart et al., 2008; Yamamura et al., 2011; Alward et al., 2014; Shevchouk et al., 2017a). Briefly, sections were sequentially rinsed 3 × 5 min in TBS, 15 min in H_2_O_2_ 3% in TBS, 3 × 5 min in TBS, and 30 min in blocking solution containing 1% BSA, 5% NGS, and 0.1% Triton X-100 in TBS. Sections were then incubated in primary antibody raised in rabbit against DCX (Abcam ab18723; 1:2000 in TBS-T, i.e., TBS containing 0.1% Triton X-100 and 1% BSA) for 1 h at room temperature and then 48 h at 4°C on a rotating shaker. Sections were washed 3 × 5 min in TBS and incubated for 2 h in the secondary antibody solution (biotinylated goat anti-rabbit antibody; Jackson ImmunoResearch 1:500 in TBS-T) at room temperature still on a rotating shaker. Sections were rinsed 3 × 5 min in TBS and incubated in the biotin-avidin complex (ABC; 1:400 Vector Elite kit, Vector Laboratories). The antigen-antibody complexes were finally visualized with the use of a SG substrate kit for peroxidase (Vector laboratories). Tissues were then mounted on microscope slides, dried and coverslipped with Eukitt mounting medium (Sigma).

### Aromatase immunohistochemistry

Sections from the third series were separated in two pools containing tissue from the telencephalon or from the diencephalon-brainstem. The telencephalon sections were immunostained for parvalbumin (PV) and chondroitin sulfate to label perineuronal nets (PNNs; see next section). Diencephalic-brainstem sections were immunostained for aromatase by methods previously described and validated (Foidart et al., 1995; Balthazart et al., 1996; Balthazart et al., 1997; Shevchouk et al., 2017b).

Briefly, sections were rinsed 3 × 5 min in TBS, 20 min in H_2_O_2_ 0.6% in TBS, 3 × 5 min in TBS, and 1 h in blocking solution containing 1% BSA, 5% NGS, and 0.2% Triton X-100 in TBS. Sections were incubated in primary antibody raised in rabbit against aromatase (a generous gift of Dr. N. Harada Toyoake, Japan; 1:10,000 in TBS-T 0.2% Triton X-100 1% BSA) for 1 h at room temperature followed by an overnight incubation at 4°C on a rotating shaker. Sections were then washed 3 × 5 min in TBS, blocked in a solution containing 1% BSA and 5% NGS and 0.2% Triton X-100 in TBS and incubated for 2 h in biotinylated goat anti-rabbit antibody (Jackson ImmunoResearch, 1:200 in TBS with 0.2% Triton X-100, 1% BSA, and 5% NGS) at room temperature on a rotating shaker. Sections were rinsed 3 × 5 min in TBS and incubated in the biotin-avidin complex ABC (1:400 Vector Elite kit, Vector Laboratories). The binding sites were finally visualized by a 10-min incubation in 0.04% 3,3’-diaminobenzidine (DAB) with 0.012% H_2_O_2_ diluted in TBS. Sections were mounted onto glass slides, dried overnight, immersed in xylene for 10 min and coverslipped with Eukitt mounting medium (Sigma).

### PV and chondroitin sulfate staining

The telencephalic tissue from the 3rd series of sections was then simultaneously immunostained for PV and chondroitin sulfate to label perineuronal nets (PNN) as described previously (Cornez et al., 2015, 2017b, 2018b) to obtain an additional measure of HVC plasticity (van 't Spijker and Kwok, 2017). Sections were rinsed 3 × 5 min in TBS and incubated in blocking solution made of 5% NGS and 0.1% Triton X-100 in TBS. Sections were then incubated overnight in a mixture of two primary antibodies including a polyclonal rabbit raised against PV (Abcam ab11427; 1:1000 in TBS-T 0.1% Triton X-100) and a monoclonal mouse anti-chondroitin sulfate antibody (1:500 in TBS-T 0.1% Triton X-100, Sigma-Aldrich C8035) for 48 h at 4°C on a rotating shaker. On the next day, sections were then washed 3 × 5 min in TBS and incubated for 2 h at room temperature on a rotating shaker in a cocktail of secondary fluorescent antibodies containing goat anti-mouse Alexa Fluor 488 (1:100, Invitrogen) and goat anti-rabbit Alexa Fluor 546 (1:200, Invitrogen). Sections were rinsed 3 × 5 min in TBS and then mounted on glass slides. Sections were dried and coverslipped with Vectashield mounting medium containing 4',6-diamidine-2'-phenylindole dihydrochloride (DAPI) to label all cell nuclei.

### Microscopy and image analysis

All quantitative analyses were performed on both sides of the brain and are presented separately taking into account whether the area under study was on the ipsilateral or contralateral side with respect to the implant targeting the POM.

#### Implant localization

The exact location of implant tips relative to the POM was checked in each subject by identifying the implant track and its end in the series of sections stained for Nissl material or immunostained for aromatase, which defines the boundaries of POM and adjacent bed nucleus of the stria terminalis (BNST) in quail (Charlier et al., 2008) and has been previously used as a marker of POM in canaries (Shevchouk et al., 2017b, 2018). These locations were then plotted on semi-schematic drawings of the canary brain derived from the published atlas (Stokes et al., 1974) where the location of the aromatase-immunoreactive (ARO-ir) cells was added based on previous immunohistochemical work on canaries (Metzdorf et al., 1999; Shevchouk et al., 2017b) and zebra finches (Balthazart et al., 1996, 1997; Saldanha et al., 2000).

#### POM volumes

All sections stained for aromatase that contained the POM in both the right and left hemispheres were photographed at 10× magnification with the Leica Application Suite 4.5.0 and a camera connected to a Leica DMRB FL 100 microscope using the same light settings for all pictures. A line was drawn around the cluster of the ARO-ir cells defining the POM identified on all sections starting from the most rostral section containing ARO-ir cells at the level of the tractus septopalliomesencephalicus to the most caudal section at the level of the anterior commissure. The area defined by this line (in µm^2^) was calculated with the area measurement function of the ImageJ software (Wayne Rasband, National Institutes of Health) and then the volume of the POM on each brain side was calculated by adding all areas and multiplying the sum by 120 μm, i.e., the distance between two successive sections in the same series.

#### HVC volumes

Photomicrographs were taken at 5× magnification of each Nissl-stained section containing HVC in both hemispheres with the same camera and microscope. HVC boundaries were drawn and its surface in each section was determined with ImageJ. These areas were added and the volume of the nucleus was obtained by multiplying this sum by 120 μm. These calculations were separately performed for both sides of the brain.

Given that HVC boundaries could also be determined by the dense cluster of DCX-ir neurons present in the nucleus, the boundaries and volume of HVC were also determined based on the sections stained for DCX by the same procedure on microphotographs taken at 10× magnification.

#### Neurogenesis and DCX quantification

In each hemisphere, cells labeled for DCX were counted in the entire HVC in all sections containing this nucleus that were used to compute the volume of the nucleus. DCX-positive cells were also counted in each of these sections in a 400 × 800 µm rectangle (0.32 mm^2^) placed at the ventral edge of HVC and another similar rectangle placed just lateral to HVC. These counts were performed on photomicrographs acquired at 5× magnification with the camera and microscope described before. The two types of DCX cells (Boseret et al., 2007; Balthazart et al., 2008) were counted separately: the fusiform cells that presumably are very young neurons still migrating and the more or less round multipolar cells that are slightly older neurons that have initiated their final differentiation. The sums of these counts of cells (fusiform and multipolar) in each location (in HVC, ventral and lateral to HVC) were computed separately and divided by the surface that had been counted to derive densities of positive cells per mm^2^.

#### PV-PNN quantification

Four separate sets of photomicrographs of HVC were obtained in each bird on the left and right side in fluorescent light at 40× magnification with a Leica DMRB FL 100 microscope, selecting in each case the four sections where HVC had the largest area. Within each set, three photomicrographs were obtained with the three different filters allowing the visualization of the Alexa Fluor 488 (green for PNN), the Alexa Fluor 546 (red for PV) and of DAPI (blue). Within each field (0.043 mm^2^) that had been photographed, we counted with ImageJ the number of PV-positive cells and the number of PNNs surrounding at least half the outline of a cell body. We additionally merged the green (PNN) and red (PV) photomicrographs to quantify the number of PNN that were surrounding PV-positive cells. We also merged the green (PNN) and blue (DAPI) photomicrographs to confirm that those PNN that were not around a PV cell were actually surrounding another type of cell. These counts were averaged across the four sections for each hemisphere of each bird, which allowed us to determine the density of PV-ir cells, of PNN, and of PNN surrounding PV-ir cells (PNN+PV) per mm^2^. This procedure also allowed us to compute the percentage of PV cells surrounded by PNN and vice versa the percentage of PNN that were located around PV cells.

### Song analysis

Songs recorded from all subjects for 2 h on days 7, 14, and 28 after placement of the brain implants were analyzed with the Raven Pro 1.5 software. Females only rarely produced long songs lasting several seconds as males typically do. Female vocalizations in most cases consisted of just a few syllables produced in rapid succession. Single syllables and very short vocalizations were very frequent and it was decided to ignore them for the present study given that they were observed with a high degree of frequency before the beginning of the steroid treatments. Instead, we focused on vocalizations lasting at least 0.4 s, separated by at least 0.4 s of silence. These vocalizations were manually selected on the sound spectrograms generated by Raven and then the program calculated a number of measures of these vocalizations including the song duration, maximum frequency, 90% bandwidth, and average Wiener entropy.

The entropy measure is an indicator of the width and uniformity of the power spectrum. It can be thought of as a measure of disorder in a sound, as a pure tone has in this context an entropy equal to zero, while higher entropy values correspond to greater disorder in a sound, as white noise would have an entropy value of 1. The average entropy reported here corresponds to the mean of all values of entropy measured for each section of the recording corresponding to songs (for the description of all these measurements, see http://www.birds.cornell.edu/brp/raven/RavenFeatures.html). From the number of songs and their duration, we additionally computed the percentage of time that birds were singing during the recordings.

### Statistics

All data associated with a single measure per subject were analyzed as appropriate by Student’s *t* tests or one-way ANOVAs with experimental groups as an independent variable. When multiple data (measures on different days or on different brain locations) were available, they were analyzed by two-way general linear model (GLM) mixed-effect analyses. All calculations were made with GraphPad Prism V8 software on MacIntosh.

HVC volumes measured in sections stained for Nissl material or for DCX were compared by the Pearson product moment correlation coefficient.

Effects were considered significant for *p* < 0.05. All data are presented by their mean ± SEM. Morphologic or histologic data from a few birds and song recordings from one subject were accidentally lost during processing resulting in a slightly smaller number of subjects for some analyses. The number of available data points is indicated in each case at the bottom of the corresponding bar in the figures.

## Results

### Implant location

Because of poor perfusion, the brain from one subject could not be used. Therefore, we were able to collect neuroanatomical data for 30 females, 18 that had been implanted with T (eight in the left, 10 in the right hemisphere) and 12 implanted with E2 (five in the left, seven in the right hemisphere).

**Figure 1.**
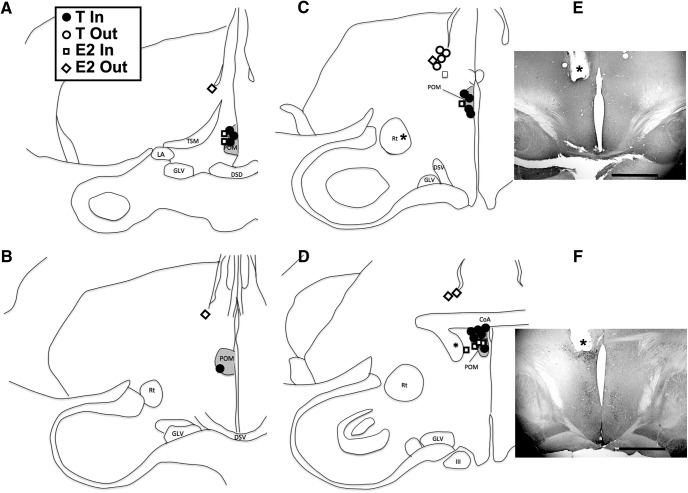
Semi-schematic maps illustrating the implant locations and their content. Panels ***A–D*** are presented in a rostral to caudal order. The inset shows the content of the implants (T or E2) and whether they were considered to be located in or out of POM. One E2 implant associated with an asterisk was considered in POM but was located in a plane caudal to the plane illustrated in ***D***. ***E***, ***F***, Photomicrographs of two brain sections immunostained for aromatase, one with an implant outside (dorsal) to POM (***E***) and one with an implant within the boundaries of the nucleus (***F***). The asterisk indicates the tip of the implant and the magnification bar is 1 mm in both cases. The induction of aromatase in the POM by T is clearly visible at the tip of the implant in ***F***. III: third nerve (nervus oculomotorius); CoA: commissura anterior; DSD: decussatio supraoptica dorsalis; DSV: decussatio supraoptica ventralis; GLV: nucleus geniculatus lateralis, pars ventralis; LA: nucleus lateralis anterior thalami; POM: medial preoptic nucleus (nucleus preopticus medialis); Rt: nucleus rotundus; TSM: tractus septopallio-mesencephalicus. *Figure Contributions*: Laura Vandries, Samar Ghorbanpoor, and Gilles Cornez performed the experiment. Laura Vandries and Jacques Balthazart analyzed the data.

Inspection of the implants tracks and tips in the Nissl-stained sections and in sections stained for aromatase revealed that out of the 18 T-implanted females, 14 (seven on the left, seven on the right side) had the tip of their implant located in the ARO-ir cell group defining the POM, while four were outside the nucleus. In the E2-implanted females, seven (one on the left, six on the right side; including one located at the very caudal end of the nucleus; Fig. 1*D*) had the tip of their implant in the POM, while five had their implant outside the nucleus.

### Data reduction

to summarize data, we first considered whether the side of T implantation (left vs right) had any impact on the results. The number of songs produced on days 7, 14, and 28 were not affected (*p* = 0.652, *p* = 0.564, and *p* = 0.659, respectively).

Similarly, we tested potential effects of the side of T implantation on all brain measures collected on both sides by two-way GLM mixed-effect analysis using the side of implantation as an independent variable and the side of measures as a repeated factor. For the measures considered (POM volume, HVC volume in DCX or Nissl-stained sections, densities of multipolar and fusiform DCX-ir neurons in, ventral or lateral to HVC, density of PNN, of PV-ir cells, of PV+PNN and percentage of PNN with PV in HVC), these analyses did not reveal significant effects of the side of implantation or of its interaction with the side of brain for all measures considered, with only two exceptions.

The analysis of POM volumes identified a significant interaction between side of implantation and the brain measure (*p* < 0.001) but no overall effect of implantation side (*p* = 0.251) or side of measure (*p* = 0.695). The volume of this nucleus was larger on the implantation side and this will be discussed in the corresponding place in the results section. In addition, analysis of the DCX-ir cells in HVC detected a significant effect of the side of implantation for multipolar cells (*p* = 0.006). This difference reflects a larger number of multipolar cells on both sides of the brain when implants were placed in the right POM compared to the left POM. This suggests that, for some unexplained reason, newborn neurons had multiplied and matured more rapidly in the group of females implanted with T on the right side. These effects will be taken into account in the following results sections, however, given the overall negative results obtained here, all subsequent analyses will only consider the pooled data as a function of whether they were collected on the ispilateral or contralateral side with respect to the steroid implant irrespective of whether implants were on the left or right side.

A similar analysis of effects of implant side was impossible for E2-implanted birds since only a single subject ended up having a cannula implanted in the left POM. Other cannulae aimed at the POM ended up outside the nucleus. The two groups of subjects were therefore pooled in this case as in the previous case.

In a second step we considered whether T-filled (*n* = 4) and E2-filled (*n* = 5) implants that ended up outside the POM had a different impact on brain and behavior. All these implants were in a position dorso-lateral to the POM and ventral to the tip of the lateral ventricle (Fig. 1). We compared all data for these two groups of Out birds by two-way GLM mixed-effect analysis with one independent factor, the two groups, and one repeated measure, the different days of recording or the two brain sides. Table 1 reports the mean ± SEM and the number of observations for each separate set of Out data, and the results of all these ANOVAs. In every single case, non-significant (*p* ≥ 0.05) probabilities were detected.

**Table 1.** Mean ± SEM and number of observations for each separate set of out data (T and E2 birds), and results of the two-way GLM mixed-effect analysis of these data (F and associated probabilities)

Variable	T out	E2 out	T vs E2	Days	Interaction
	Mean ± SEM (*n*)	Mean ± SEM (*n*)	*F*, *p*	*F*, *p*	*F*, *p*
Number of songs D7	27.7 ± 17.6 (4)	122 ± 57 (5)			
D14	37.5 ± 30.8 (4)	83.8 ± 38.9 (5)			
D28	31.7 ± 12.3 (4)	17.0 ± 11.9 (5)	*F* = 1.204, *p* = 0.308	*F* = 1.783, *p* = 0.216	*F* = 1.961, *p* = 0.177
Song duration D7	0.34 ± 0.11 (4)	0.28 ± 0.12 (5)			
D14	0.35 ± 1.12 (4)	0.39 ± 0.10 (5)			
D28	0.44 ± 0.02 (4)	0.28 ± 0.11 (5)	*F* = 0.245, *p* = 0.629	*F* = 0.303, *p* = 0.674	*F* = 0.809, *p* = 0.465
% Time singing D7	0.41 ± 0.22 (4)	1.61 ± 0.77 (5)			
D14	0.53 ± 0.38 (4)	1.13 ± 0.52 (5)			
D28	0.40 ± 0.17 (4)	0.22 ± 0.15 (5)	*F* = 1.123, *p* = 0.324	*F* = 2.013, *p* = 0.187	*F* = 1.792, *p* = 0.203
Maximum frequency D7	3553 ± 328 (3)	4095 ± 239 (3)			
D14	3580 ± 342 (3)	3727 ± 439 (4)			
D28	3972 ± 403 (4)	4176 ± 213 (3)	*F* = 0.189, *p* = 0.678	*F* = 0.682, *p* = 0.484	*F* = 0.519, *p* = 0.614
Bandwidth D7	1104 ± 167 (3)	1885 ± 834 (3)			
D14	1128 ± 97(3)	2156 ± 1185 (4)			
D28	947 ± 141 (4)	1268 ± 329 (3)	*F* = 1.036, *p* = 0.348	*F* = 0.499, *p* = 0.612	*F* = 0.349, *p* = 0.715
Mean entropy D7	2.98 ± 0.21 (3)	3.29 ± 0.39 (3)			
D14	2.85 ± 0.24 (3)	2.93 ± 0.15 (4)			
D28	2.74 ± 0.16 (4)	2.92 ± 0.10 (3)	*F* = 0.524, *p* = 0.496	*F* = 4.037, *p* = 0.128	*F* = 1.875, *p* = 0.245
Variable	T out	E2 out	T vs E2	ipsilateral-contralateral	Interaction
POM volume ipsilateral contralateral	0.06 ± 0.01 (4) 0.05 ± 0.01 (4)	0.02 ± 0.02 (3) 0.02 ± 0.01 (3)	*F* = 3.022, *p* = 0.142	*F* = 0.811, *p* = 0.408	*F* = 0.227, *p* = 0.653
HVC volume Nissl ipsilateral contralateral	0.10 ± 0.02 (4) 0.11 ± 0.02 (4)	0.14 ± 0.02 (4) 0.12 ± 0.02 (4)	*F* = 0.810, *p* = 0.403	*F* = 0.516, *p* = 0.499	*F* = 1.507, *p* = 0.265
HVC volume DCX ipsilateral contralateral	0.11 ± 0.03 (4) 0.10 ± 0.02 (4)	0.10 ± 0.02 (5) 0.10 ± 0.01 (5)	*F* = 0.053, *p* = 0.824	*F* = 1.032, *p* = 0.343	*F* = 0.002, *p* = 0.967
Fusiform DCX in HVC ipsilateral contralateral	86.2 ± 12.3 (4) 64.5 ± 10.7 (4)	56.0 ± 6.9 (5) 53.6 ± 4.6 (5)	*F* = 3.559, *p* = 0.101	*F* = 5.143, *p* = 0.058	*F* = 3.302, *p* = 0.112
Multipolar DCX in HVC ipsilateral contralateral	152.0 ± 35.2 (4) 142.0 ± 30.7 (4)	151.4 ± 9.7 (5) 144.4 ± 8.0 (5)	*F* = 0.001, *p* = 0.976	*F* = 1.715 *p* = 0.232	*F* = 0.053, *p* = 0.824
Fusiform DCX vtr. HVC ipsilateral contralateral	11.2 ± 3.1 (4) 10.0 ± 2.6 (4)	7.8 ± 0.8 (5) 7.8 ± 2.6 (5)	*F* = 1.099, *p* = 0.329	*F* = 0.110, *p* = 0.750	*F* = 0.110, *p* = 0.750
Multipolar DCX vtr. HVC ipsilateral contralateral	19.0 ± 3.8 (4) 18.5 ± 2.6 (4)	23.0 ± 2.8 (5) 21.4 ± 2.1 (5)	*F* = 1.255, *p* = 0.300	*F* = 0.161, *p* = 0.700	*F* = 0.044, *p* = 0.839
Fusiform DCX lat. HVC ipsilateral contralateral	13.0 ± 3.5 (4) 13.2 ± 2.5 (4)	7.4 ± 0.7 (5) 10.8 ± 2.4 (5)	*F* = 1.848, *p* = 0.216	*F* = 1.401, *p* = 0.275	*F* = 1.043, *p* = 0.341
Multipolar DCX lat. HVC ipsilateral contralateral	8.0 ± 1.3 (4) 6.5 ± 1.0 (4)	7.8 ± 1.5 (5) 8.6 ± 1.3 (5)	*F* = 0.369, *p* = 0.562	*F* = 0.123, *p* = 0.736	*F* = 1.332, *p* = 0.286
PNN density ipsilateral contralateral	36.7 ± 7.0 (3) 58.0 ± 22.1(3)	34.8 ± 20.4 (5) 46.4 ± 22.6 (5)	*F* = 0.070, *p* = 0.800	*F* = 0.835, *p* = 0.396	*F* = 0.072, *p* = 0.796
PV-ir density ipsilateral contralateral	106.3 ± 17.1 (3) 104.0 ± 0.0 (3)	116.6 ± 17.6 (5) 143.0 ± 25.7 (5)	*F* = 1.289, *p* = 0.278	*F* = 0.308, *p* = 0.589	*F* = 0.439, *p* = 0.520
PV+PNN density ipsilateral contralateral	6.0 ± 3.5 (3) 11.3 ± 3.9 (3)	7.6 ± 6.4 (5) 10.4 ± 4.6 (5)	*F* = 0.003, *p* = 0.957	*F* = 0.618, *p* = 0.462	*F* = 0.059, *p* = 0.815
% PNN with PV ipsilateral contralateral	21.3 ± 14.9 (3) 22.7 ± 5.4 (3)	2.2 ± 2.2 (5) 44.4 ± 23.1 (5)	*F* = 0.006, *p* = 0.938	*F* = 1.744, *p* = 0.213	*F* = 1.537, *p* = 0.238

Therefore, in the rest of this presentation, all results are analyzed after being pooled in 3 experimental groups: birds with T in POM (T group; *n* = 14), E2 in POM (E2 group; *n* = 7), and birds with T or E2 outside of POM (Out group; *n* = 9)

### Morphologic data

**Figure 2.**
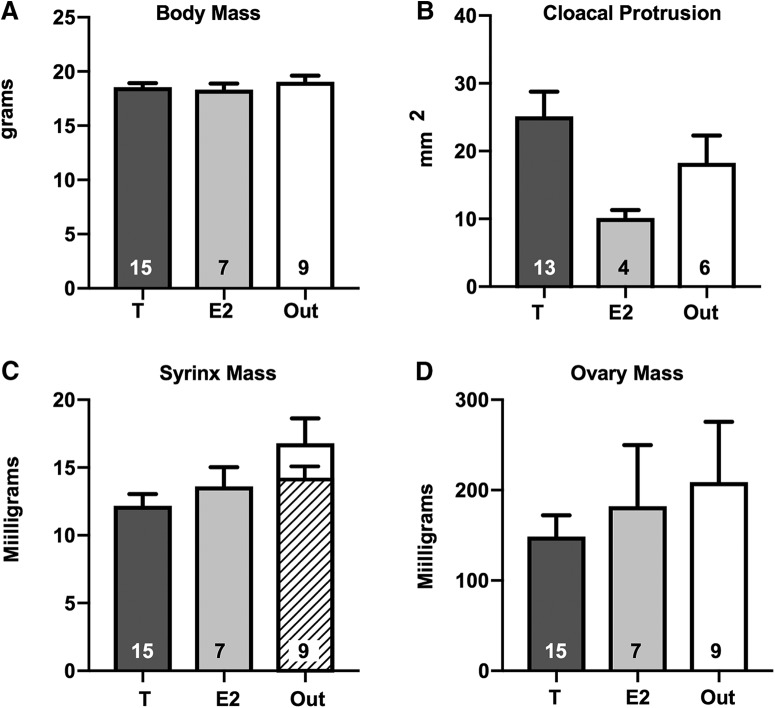
Mean ± SEM of all morphologic measures collected in the three groups of females at the end of the experiment. No significant difference could be detected among the three groups in body mass (***A***), cloacal protrusion (***B***), nor ovary mass (***D***) except for syrinx mass (***C***), but this difference disappears when two outliers in the Out group are removed (hatched bar; see text). The number of available data points is indicated in each case at the bottom of the corresponding bar. *Figure Contributions*: Laura Vandries, Samar Ghorbanpoor, Gilles Cornez, and Olesya Shevchouk performed the experiment. Laura Vandries and Jacques Balthazart analyzed the data.

At the end of the experiment, the body mass of the three groups of females was very similar (*F*_(2,30)_ = 0.478, *p* = 0.625; Fig 2*A*). The cloacal protrusion, a marker of androgen action (Luine et al., 1980; Tramontin et al., 2000) was on average slightly increased in the T group and decreased in the E2 group by comparison with the control Out group (Fig. 2*B*) but the effect was not statistically significant (*F*_(2,20)_ = 2.875, *p* = 0.080). Surprisingly syrinx mass differed between groups (*F*_(2,28)_ = 3.516, *p* = 0.043; Fig. 2*C*) with the T group being significantly smaller (*p* = 0.034) than the Out group (*p* = 0.034). This effect might, however, only result from a poor (too large) dissection in two subjects of the Out group that were clearly outliers (25.5 and 26.2 mg vs a mean ± SD of 14.21 ± 2.15 after their exclusion). If these two values are excluded (Fig. 2*C*, hatched bar) there is no longer an effect of treatments on syrinx weight (*F*_(2,28)_ = 1.109, *p* = 0.345). Ovary mass was also not affected by the treatments (*F*_(2,28)_ = 0.471, *p* = 0.629; Fig. 2*D*).

### Singing behavior

**Figure 3.**
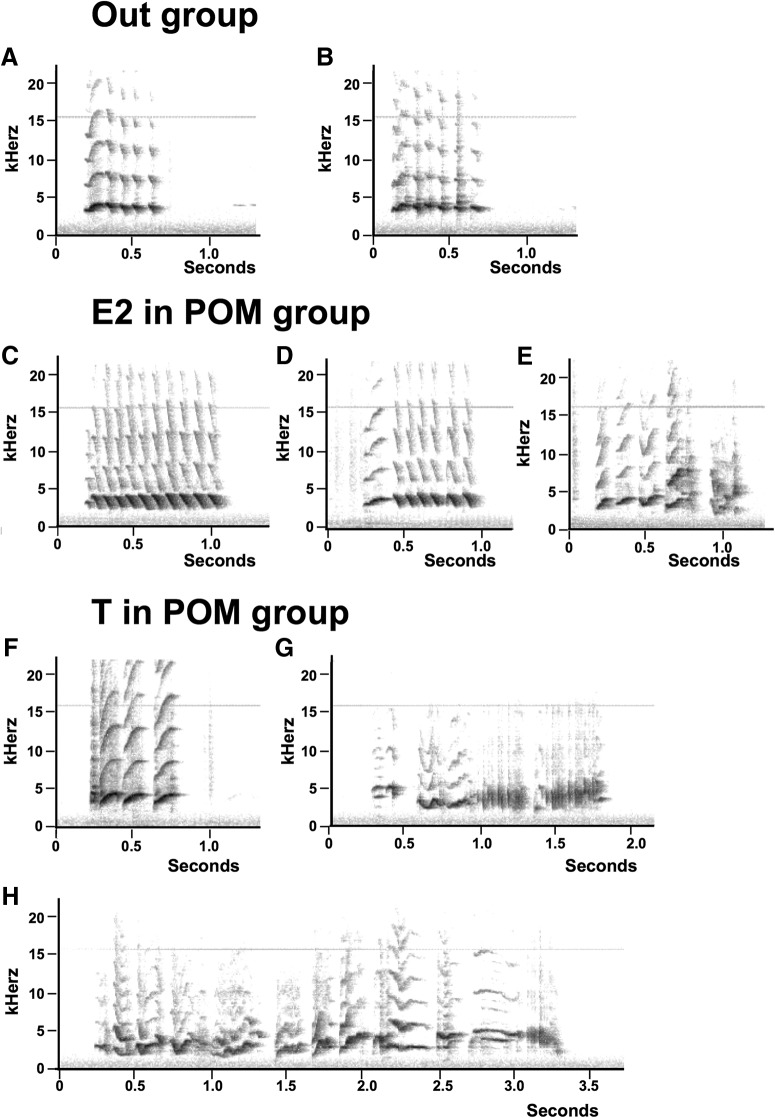
Representative sonograms illustrating the songs produced by females treated with T or E2 implanted in or out of POM. Birds in the Out group only produced very short songs, usually consisting in the repetition of a single syllable (***A***, ***B***). E2 (***C–E***) or T (***F–H***) implanted in POM increased the duration of some but not all songs that consisted in some cases of multiple syllables. ***H***, One of the most complex songs seen in the T in POM groups. *Figure Contributions*: Laura Vandries, Gilles Cornez, and Jacques Balthazart analyzed the data.

Most females were at the beginning of the experiment producing short vocalizations including only one or two syllables that lasted only 0.2–0.4 s. Within 7 d after implantation of T or E2, these vocalizations became more frequent and they increased based both on duration and on the number of different syllables present within a song. The maximal rate of production was observed on day 14 in the T group and on day 28 in the E2 group. Figure 3 illustrates the type of songs that were produced by T or E2-treated females with implants in POM as well as by a female with an implant that missed its target.

**Figure 4.**
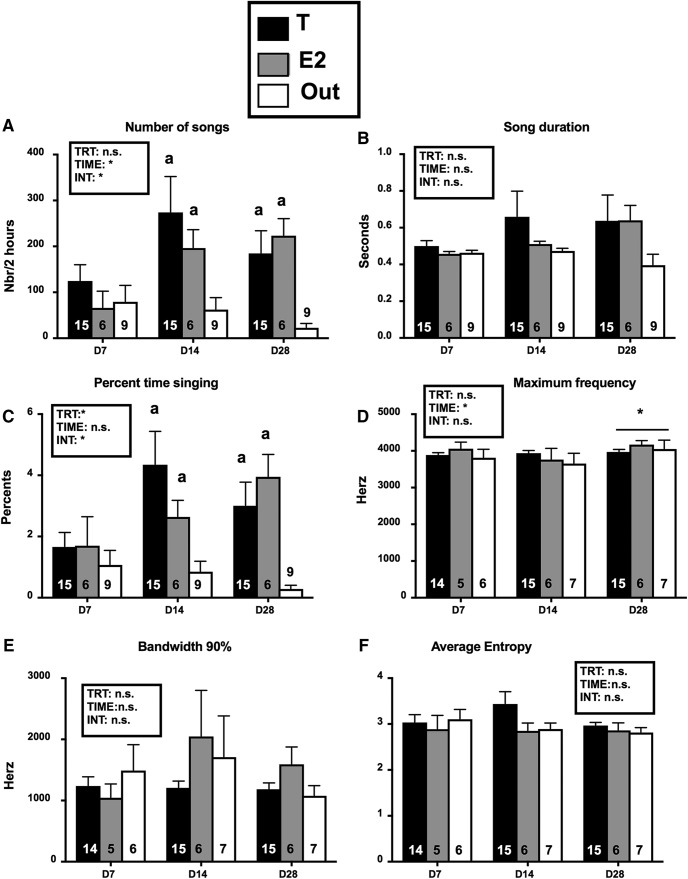
Summary of all measures of songs produced during 2 h of recording on days (d) 7, 14, and 28 after implantation of the steroids in the brain. The figure shows from top left to bottom right the number of songs per recording session of 2 hours (***A***), the average duration of these songs (***B***), the percentage of time that females spent singing (***C***) and then the maximum frequency (***D***), the 90% range of the bandwith (***E***) and the average entropy (***F***) of these songs. Data were analyzed by two-way GLM mixed-effect analysis with the three groups as independent factor and the three recording days as repeated factor and the results are schematically reported above each graph (Trt, treatment; time, time after implantation; Int, interaction; *p < 0.05). Significant effects were followed by *post hoc* Tukey tests whose results are indicated by letters above the bars (a = *p* < 0.05 compared to the corresponding Out group). The asterisk above a bar refers to time effects and indicates a significant difference with the D7 point. The number of available data points is indicated in each case at the bottom of the corresponding bar. *Figure Contributions*: Laura Vandries and Jacques Balthazart analyzed the data.

The visual inspection of all sonograms indicated that, as illustrated in Figure 3, there was a large variation in the duration and structure of these songs. Some lasted a very short time and consisted of the repetition of a single syllable; others had multiple syllable types that were repeated for durations up to 6–7 s. This variability is reflected in the large variability of durations illustrated in Figure 4*B*.

All songs identified during the 2-h recording sessions that occurred on days 7, 14, and 28 of the experiment were quantitatively evaluated with the Raven Pro software and results were analyzed by two-way GLM mixed-effect analysis with the three different groups and three recording times as independent and repeated factors, respectively. The number of songs produced (Fig. 4*A*) significantly varied over time (*F*_(1.95, 52.64)_ = 4.304, *p* = 0.019) and these changes were different in the three groups as revealed by a significant interaction between time and groups (*F*_(4,54)_ = 2.780, *p* = 0.036). The overall group difference was, however, not statistically significant (*F*_(2,27)_ = 2.760, *p* = 0.081). Comparisons of the T and E2 groups to the Out group by the Tukey test indicated significant differences between T and Out and between E2 and Out on days 14 and 28.

The average duration of individual songs (Fig. 4*B*) slightly increased over time and did so on average more prominently in the T and E2 groups but analysis of these data indicated no significant effect of time (*F*_(1.15,27.12)_ = 0.717, *p* = 0.424), no group difference and no interaction (*F*_(2,26)_ = 0.797, *p* = 0.461 and *F*_(4,47)_ = 0.685, *p* = 0.606, respectively).

The percentage of time that birds were singing during the 2-h recordings (Fig. 4*C*) that reflects both the numbers of songs and their duration also increased over time although the effect was not fully significant (*F*_(1.94,52.30)_ = 3.087, *p* = 0.056). There was, however, a significant overall group difference (*F*_(2,27)_ = 3.924, *p* = 0.032) and an interaction between groups and time (*F*_(4,54)_ = 3.192, *p* = 0.020). Tukey multiple comparisons tests confirmed the presence of significant differences between T and Out and between E2 and Out on days 14 and 28.

A more detailed analysis of the songs sampled focused on three additional parameters: the song maximum frequency, the 90% bandwidth, and the average entropy. Analyses of these measures by mixed-effects model (birds that were not singing on a given day could not be assigned a value) revealed no group difference (*p* ≥ 0.317) and no interaction (*p* ≥ 0.291). A moderate time effect was observed for the analysis of the maximum frequency (*F*_(1.49,34.47)_ = 3.759, *p* = 0.045), but not for the two other measures. Yet, since it is not associated with an interaction, this effect cannot result from the steroid treatments. *Post hoc* tests indicated that the song maximal frequency was significantly higher on day 28 than on day 7.

### POM volume

**Figure 5.**
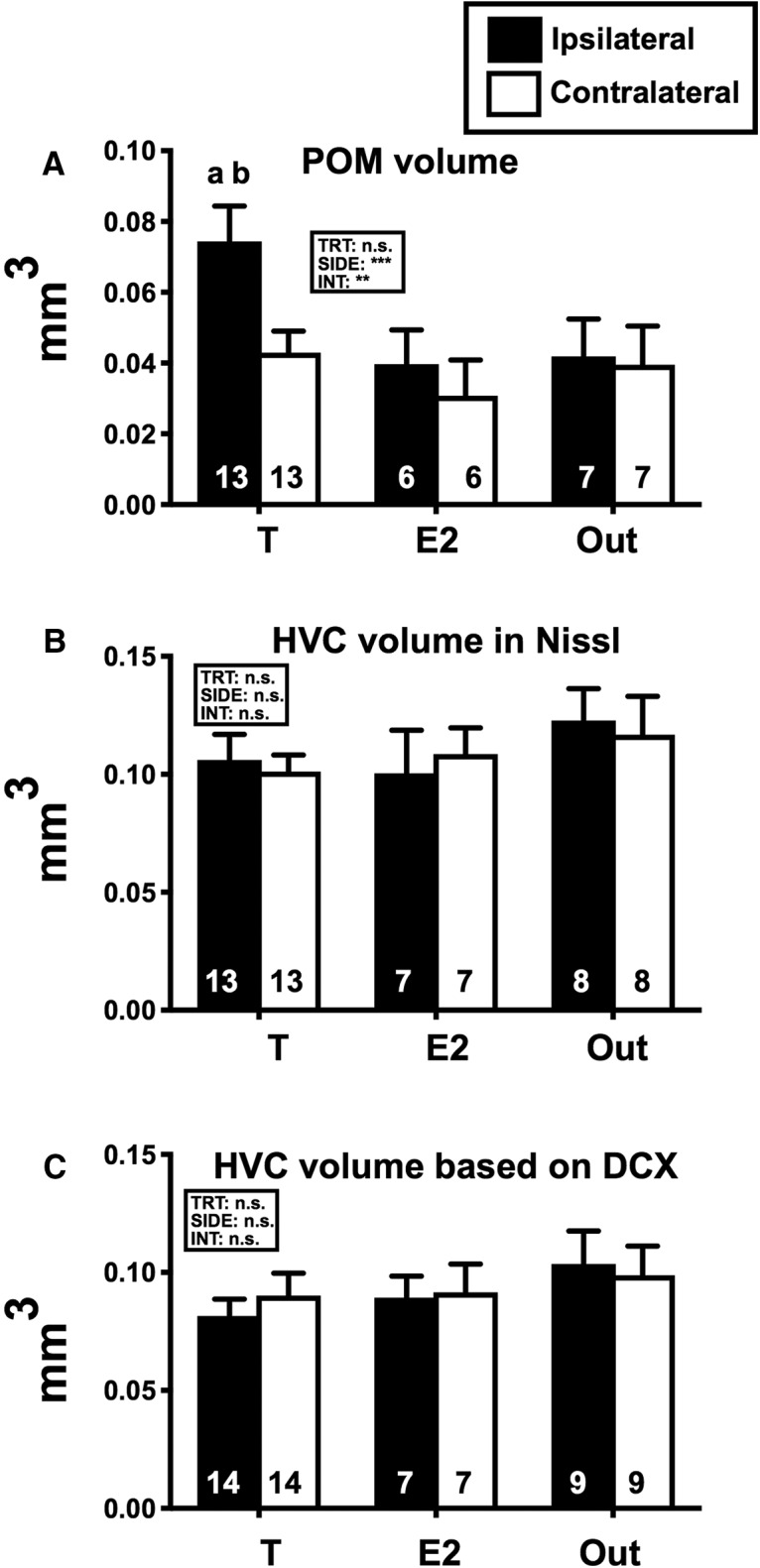
Mean (±SEM) volumes of the POM as identified by the dense cluster of ARO-ir cells (***A***), and of nucleus HVC as identified in Nissl-stained sections (***B***) and by the dense cluster of DCX-ir cells (***C***) in females treated with T or E2 implanted in or out of POM on the ipsilateral (left bar in each pair) or contralateral (right bar in each pair) side. Data were analyzed by two-way GLM mixed-effect analysis with the three groups as independent and the two sides of the brain as repeated factor and the results are schematically reported above each graph (TRT, treatment; SIDE, brain side with respect to the implant; INT, interaction; **p < 0.01, ***p < 0.001). Results of Tukey *post hoc* tests comparing the three groups on each brain side are indicated by letters (a,b = *p* < 0.05 compared to the Out and E2 group, respectively, on the same brain side). The number of available data points is indicated in each case at the bottom of the corresponding bar. *Figure Contributions*: Laura Vandries, Samar Ghorbanpoor, and Olesya Shevchouk performed the experiment. Laura Vandries and Jacques Balthazart analyzed the data.

**Figure 6.**
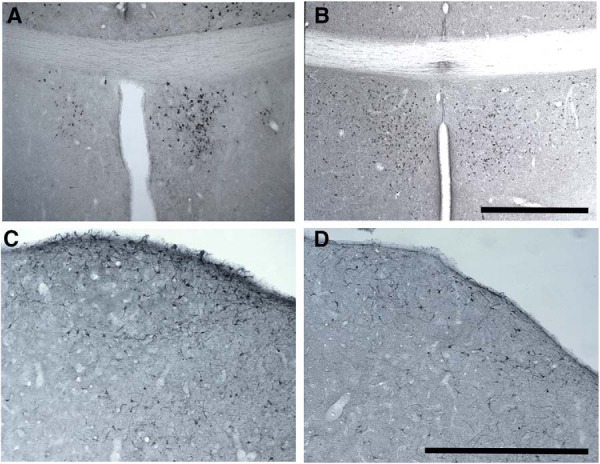
Representative photomicrographs of the preoptic area (***A***, ***B***) or of the song control nucleus HVC (***C***, ***D***) illustrating the main experimental effects. Panels in the top row show the preoptic area stained for aromatase in a female with a T implant on the right side showing the aromatase induction (***A***) or in a female of the Out group showing basal aromatase expression (***B***). Panels in the bottom row show nucleus HVC stained for DCX in a female with a T implant in POM (***C***) and an Out bird (***D***) illustrating the increase by T in POM of the density of DCX-ir cells in HVC. Magnification bars are 500 µm in both cases and refer to both panels on the same row. *Figure Contributions*: Laura Vandries and Samar Ghorbanpoor performed the experiment. Laura Vandries and Jacques Balthazart analyzed the data.

The volume of the POM as defined by the dense group of ARO-ir neurons (Fig 6*A-B*) was analyzed by a two-way GLM mixed-effect analysis with the three groups as independent factors and the two sides of the brain (ipsilateral and contralateral to the implant) as a repeated factor. This analysis revealed a significant effect of the brain side (*F*_(1,23)_ = 16.87, *p* < 0.001) and interaction between groups and side of the brain (*F*_(2,23)_ = 8.241, *p* = 0.002) but no overall effect of treatments (*F*_(2,23)_ = 1921, *p* = 0.169; Fig 5*A*). The Tukey multiple comparisons tests indicated that POM volume was larger on the implantation side in the T group compared to both the E2 and Out group but these differences was not present on the contralateral side.

### HVC volume

HVC volume was assessed both in Nissl-stained sections and in sections stained for DCX that highlights the boundaries of HVC based on the higher density of DCX-ir cells inside as compared to outside the nucleus (Fig. 6*C-D*). The two-way GLM mixed-effect analysis identified no effect of treatment (Nissl: *F*_(2,25)_ = 0.574, *p* = 0.571; DCX: *F*_(2,27)_ = 0.672, *p* = 0.519), no difference between ipsilateral and contralateral sides (Nissl: *F*_(1,25)_ = 0.042, *p* = 0.838; DCX: *F*_(1,27)_ = 0.182, *p* = 0.673) and no interaction between these factors (Nissl: *F*_(2,25)_ = 0.912, *p* = 0.415; DCX: *F*_(2,27)_ = 0.788, *p* = 0.465).

**Figure 7.**
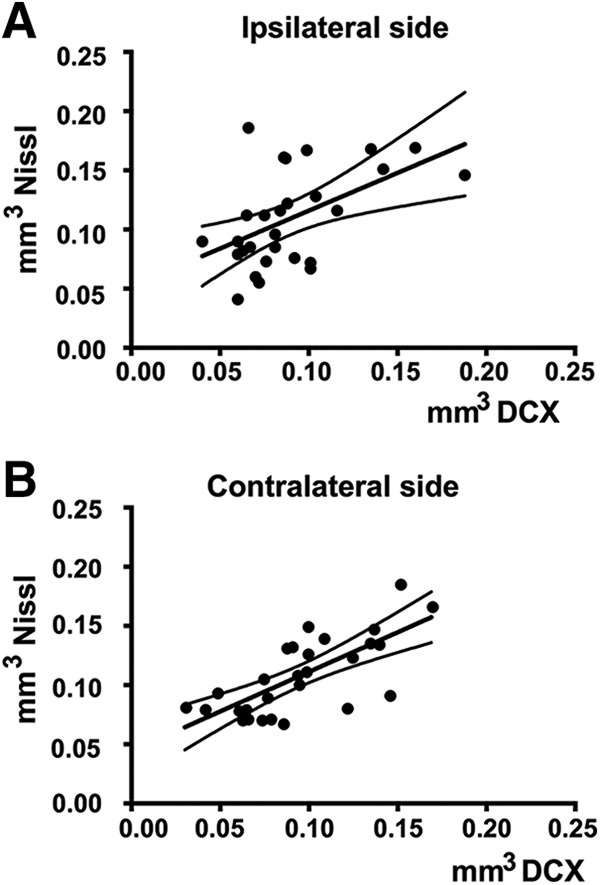
Correlation between the volumes of HVC as measured in Nissl-stained sections (***B***) and by the dense cluster of DCX-ir cells. Data were separately analyzed for volumes measured on the side ipsilateral or contralateral side to the steroid implants. The graph illustrates the significant regression line and the 95% confidence intervals. *Figure Contributions*: Laura Vandries and Jacques Balthazart analyzed the data.

Interestingly, the volumes of HVC as measured in Nissl-stained or DCX-ir-stained sections were significantly correlated both on the ipsilateral and contralateral sides, even if this correlation was not perfect (ipsilateral: *r* = 0.520, *p* = 0.005; contralateral: *r* = 0.717, *p* < 0.001; Fig. 7).

### Neurogenesis (DCX)

**Figure 8.**
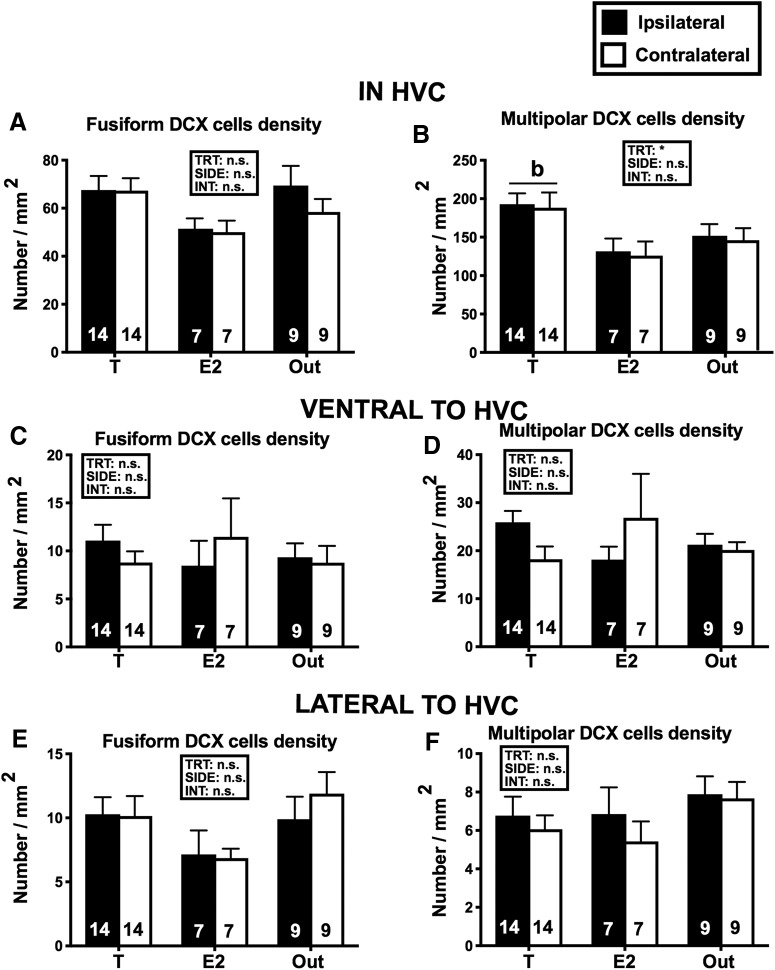
Mean (±SEM) densities (numbers/mm^3^) of fusiform (***A***, ***C***, ***E***) and multipolar (***B***, ***D***, ***F***) DCX-ir cells in HVC (***A***, ***B***) and in area directly ventral (***C***, ***D***) or lateral (***E***, ***F***) to this nucleus in the three experimental groups on the brain side ipsilateral and contralateral to the steroid implants. Data were analyzed by two-way GLM mixed-effect analysis with the three groups as independent and the two sides of the brain as repeated factor and the results are schematically reported above each graph (TRT, treatment; SIDE, brain side relative to implant; INT, interaction; *p < 0.05). Significant effects of treatments were followed by Tukey *post hoc* tests whose results are expressed as follows: b = *p* < 0.05 by comparison with the E2 group. The number of available data points is indicated in each case at the bottom of the corresponding bar. *Figure Contributions*: Laura Vandries, Samar Ghorbanpoor, and Olesya Shevchouk performed the experiment. Laura Vandries and Jacques Balthazart analyzed the data.

Despite the absence of global effect of the treatments on the volume of HVC, we asked whether steroids implanted in POM had affected the rate of neurogenesis in this nucleus. Fusiform and multipolar DCX-ir cells were therefore quantified separately in HVC and, as a control, in two equivalent areas, one just ventral and one just lateral to the nucleus (Fig. 8).

Two-way GLM mixed-effect analysis of the number of DCX-ir cells in HVC (three treatments as independent factor and two sides, ipsilateral vs contralateral, as repeated factor) identified a statistical trend suggesting an effect of the treatments on fusiform (fusiform: *F*_(2,27)_ = 2.527, *p* = 0.099**)** and a significant effect of treatments on multipolar (*F*_(2,27)_ = 3.658, *p* = 0.039) DCX-ir cells (Fig. 8*A*,*B*). There was no effect of brain side (fusiform: *F*_(1,27)_ = 1.208, *p* = 0.281; multipolar: *F*_(1,27)_ = 0.737, *p* = 0.399**)** and no interaction between brain side and treatment (fusiform: *F*_(2,27)_ = 0.825, *p* = 0.449; multipolar: *F*_(2,27)_ = 0.024, *p* = 0.976). The overall treatment effect of multipolar cells was associated in the Tukey *post hoc* tests with a significant difference between the T and E2 group (*p* = 0.049) but the T versus Out difference failed to reach statistical significance (*p* = 0.164).

Note that in the data reduction section, we had noticed that females with a T implant in the right POM had more multipolar DCX-ir cells in HVC. Given, however, that identical numbers of birds had an implant in the left and in the right POM this difference based on side of implantation has no impact on the results presented here. There was actually no average difference in numbers of cells between the ipsilateral and contralateral sides of the brain with respect to the implant.

Similar analyses of DCX-ir cells densities counted in an equivalent area just ventral or just lateral to HVC (Fig. 8*C–F*) identified no effect of treatments (*p* ≥ 0.141), of the side of the brain (*p* ≥ 0.273) and of their interaction (*p* ≥ 0.672, except for the multipolar DCX-ir cells in ventral position where *p* = 0.070 but this effect does not seem to be associated with an interpretable effect of the steroids; detailed statistics not shown).

### PNNs

This experiment was also providing an occasion to probe the mechanisms underlying the T-induced expression of PNN in the song control system. Previous work in male canaries demonstrated that systemic treatment with exogenous T increases the density of PNN in HVC (Cornez et al., 2017a). Given that this treatment simultaneously activated an intense singing activity, it was impossible in this situation to determine whether the increased PNN expression results from a direct action of T on HVC or indirectly from the increased neuronal activity in this nucleus. Females receiving a T implant in POM potentially allowed us to discriminate between these two possibilities.

**Figure 9.**
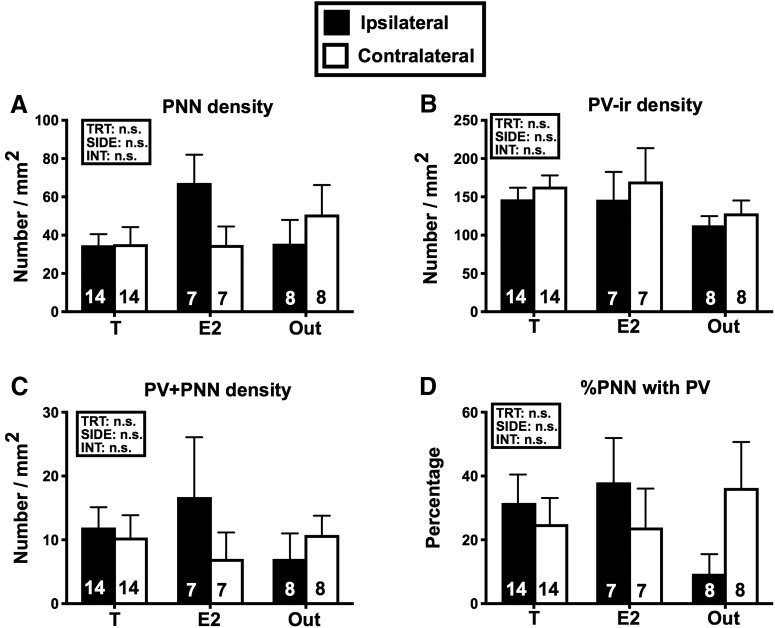
Mean (±SEM) densities (numbers/mm^3^) of PNN (***A***), of PV-ir cells (***B***), of PV-ir cells surrounded by PNN (***C***) and percentage of PNN present around PV-ir cells (***D***) in the three experimental groups on the side ipsilateral and contralateral side to the steroid implants. Data were analyzed by two-way GLM mixed-effect analysis with the three groups as independent and the two sides of the brain as repeated factor and the results are schematically reported above each graph (TRT, treatment; SIDE, brain side relative to implant; INT, interaction). No significant effect was detected. The number of available data points is indicated in each case at the bottom of the corresponding bar. *Figure Contributions*: Laura Vandries and Gilles Cornez performed the experiment. Laura Vandries and Jacques Balthazart analyzed the data.

The density of PNN (number per mm^2^) in HVC was not affected by the treatments (*F*_(2,26)_ = 0.947, *p* = 0.401), side of the brain (*F*_(1,26)_ = 0.489, *p* = 0.490) or their interaction (*F*_(2,26)_ = 2678, *p* = 0.087; Fig. 9*A*). Since most PNN form around PV-positive cells, this type of cells was also quantified but this identified no significant effect (treatments: *F*_(2,26)_ = 1.301, *p* = 0.289; side: *F*_(1,26)_ = 1.285, *p* = 0.267; interaction: *F*_(2,26)_ = 0.019, *p* = 0.980). The density of PV-positive cells associated to PNN was similarly not affected (treatments: *F*_(2,52)_ = 0.203, *p* = 0.817; side: *F*_(1,52)_ = 0.438, *p* = 0.511; interaction: *F*_(2,52)_ = 0.895, *p* = 0.415) and this was also the case of the percentage of PNN associated with PV cells (treatments: *F*_(2,52)_ = 0.260 *p* = 0.778; side: *F*_(1,52)_ = 0.051, *p* = 0.821; interaction: *F*_(2,52)_ = 1.844, *p* = 0.168).

## Discussion

This experiment demonstrates that, as shown previously in males (Alward et al., 2013, 2016), implantation of T in the POM increases vocal production in female canaries. This behavioral effect was accompanied by changes in aromatase expression in the POM and also by a bilateral increase in neurogenesis in HVC. No change in PNN expression, which is usually associated with song crystallization in both canaries and zebra finches, was, however, observed in HVC. Because effects of T on singing are thought to be induced at least in part by the action of its estrogenic metabolites at the cellular level (Fusani et al., 2003; Fusani and Gahr, 2006), we also implanted some females with E2 in the POM and demonstrate that this resulted in relatively similar behavioral effects, but there were no statistically significant effects when one examined the neural measures. No significant difference between treatment groups could be detected in body mass, the size of the cloacal protrusion (an androgen-dependent structure), the mass of the syrinx (androgen-dependent also) or of the ovary. The syrinx mass in particular was roughly similar to what was previously observed in females that are not systemically treated with sex steroids (Shevchouk et al., 2017b). These data suggest that there was little or no leakage of steroids from the brain implants to the periphery and at any rate that this leakage was not differential between the three groups of subjects and thus cannot explain differences among treatment groups. This conclusion is also supported by the observation that T implants increased POM volume on the ipsilateral but not on the contralateral side of the brain, indicating that steroid diffusion did not even reach his adjacent location. These results allow us to draw a number of general conclusions but also raise a number of questions that need to be considered.

### Singing activity

In the large number of subjects that received a T or E2 implant in the POM, a clear increase in singing activity was detected. This was reflected in the production of a larger number of vocalizations and, in some subjects, an increase in their duration, but this latter effect was too variable to be significant. The percentage of time spent singing that reflects both the number and duration of these vocalizations was also markedly increased by both T and E2 implants, when located in the POM as compared to birds in which the implant had missed its target.

*Post hoc* tests indicated that a significant effect of T acting in the POM on singing behavior was observed earlier after treatment than for E2 (day 14 vs 28), while the reverse would be expected if all effects of T are mediated after its conversion to E2. This observation could thus support the idea that T itself is implicated in the activation of singing, but the average difference between these two groups was small and could simply reflect slightly different localizations of the implants, a differential diffusion of the steroid in brain tissue or even the lower statistical power of the experiment for the E2 group (7 E2 in POM vs 14 T in POM females).

The quality of the songs produced by these females remained very poor as compared to male-typical songs. Their average duration barely increased, with only a few females producing songs lasting longer than one and even more rarely two seconds. No significant effect of treatments on maximum frequency, bandwidth, entropy, or average entropy could be observed. This pattern corresponds to a large extent to what was observed in males, where implantation of T in the POM increased the song rate, but did not modify the quality of the vocalizations (Alward et al., 2013, 2016).

Songs in females with T or E2 in POM were, however, of much poorer quality than in similarly treated males. Average song duration in males with T in POM was indeed around 4 s (Alward et al., 2013), while it barely reached 0.6 s in females. Furthermore, female songs usually consisted of the repetition of two or three syllables that were not fully crystallized (no sharp definition in sonograms, variability from one rendition to the next), while more diversity in syllable usage was observed in males with T in POM even if a large degree of variability between successive renditions was also present.

Overall, the female songs observed here had a distribution of energy that showed a higher degree of general disorder than fully crystallized male songs. In two independent unpublished experiments performed in our laboratory on the same breed of canaries, we indeed observed that the average entropy of male songs in the spring is around 2.5, while entropy measured here was ≥3 (Cornez et al., 2018a; Cornez and Balthazart, unpublished data).

The origins of these sex differences in response to hormone treatment are difficult to identify at this stage. It is, however, likely that it reflects a rather fundamental difference between males and females since even when treated systemically with T for three weeks males and females still sing songs that are qualitatively different (Madison et al., 2015). It is unlikely that the difference between songs observed here in females and those previously observed in similarly treated males (Alward et al., 2013, 2016) simply reflects a difference in hormonal activation. The size and position of implants used here are indeed similar to those used and observed in the male experiments. One possible reason for this difference is that the females receiving these POM implants have not experienced as robust a process of sensorimotor song learning as the males experienced. It is known that female songbirds can learn to recognize the songs of their conspecific males (Gentner and Hulse, 2000; Catchpole and Slater 2008; Nowicki and Searcy, 2014). However, it is reasonable to assume that the hormonal activation of song in an individual who has not robustly experienced sensorimotor learning would be less effective than in an individual who has. This sex difference could of course also reflect more fundamental genetic sex differences related to song production in canaries, but this could only be determined by ontogenetic experiments investigating the development of song in males and females exposed to identical endocrine conditions.

It should also be noted that a number of song features significantly changed over the course of the experiment, but in a similar manner in the three groups of subjects (no effect of treatments and no interaction of time with treatment). This is the case for the maximum frequency and the three measures of song amplitude (maximum, peak, and RMS) that are not reported here. These changes presumably reflect the transfer from short to long days (from 8 to 16 h of light per day) of the birds at the beginning of the experimental phase that should have promoted a limited increase in ovarian activity and consequently in circulating E2 concentrations.

### The POM as identified by aromatase immunohistochemistry

The position of implants was mapped in sections stained for Nissl material but also stained by immunohistochemistry for aromatase, which provides a clearer and easier identification of the POM. It was shown previously that a systemic treatment with T increases within a few days aromatase expression and the related POM volume as assessed by the dense cluster of ARO-ir cells in female (Shevchouk et al., 2017b) and male (Shevchouk et al., 2019) canaries.

A significantly larger volume of the ARO-ir cell group defining POM was observed here on the side ipsilateral to the brain implant in the T group, but a similar effect was not observed after implantation of E2. This increase specifically observed in the ispilateral side of T birds confirms the local efficacy of the steroid implants in the present design and, as already mentioned, their action limited to the immediate surrounding of the implant tip. It has previously been shown in several avian species that E2 largely mimics the effects of T in the induction of aromatase (Hutchison and Steimer, 1986; Hutchison et al., 1989; Harada et al., 1993). Why this was not the case here remains unexplained and can only be ascribed at this point to the dose or diffusion of the steroid.

### HVC volume and neurogenesis

It was previously observed that unilateral implantation of T in the POM of males significantly increases HVC volume on both sides of the brain (Alward et al., 2013, 2016), but this effect was not replicated here in females. Volumes measured both in Nissl-stained sections and based on the dense DCX-ir cell group identified no treatment effect and no treatment by side interaction, although these two sets of measures were very significantly correlated suggesting that the two labels identify the same structure.

In males with a T implant in POM, analysis of the relationship between HVC volume and singing activity had suggested that the increased volume is in part activity dependent, although local actions of T also participate to the increase in HVC volume as observed in birds which additionally had a T implant near HVC (Alward et al., 2016). Since the amount of T implanted here was similar to the amount implanted in the published male experiments, it can be suspected that the singing activity induced here in females was not intense enough to promote a detectable growth of HVC. Accordingly in this experiment, in contrast to what was observed before in males, no correlation was detected between the number of songs or percentage time spent singing and the measures of HVC volumes (ipsilateral or contralateral side, Nissl-stained or DCX-ir cell group; –0.270 ≤ *R* ≤ 0.034; *p* = 0.157 for the largest negative value, *p* ≥ 0.812 otherwise). This growth might alternatively be slower in females than in males and a longer exposure to the steroids may have produced significant effects.

Surprisingly, however, a significant increase in multipolar DCX-ir cells was observed in the HVC of T birds, while no difference was detected ventral or lateral to HVC. These cellular changes were obviously not sufficient to modify the overall volume of HVC, but they clearly demonstrate that steroid implantation in POM affected the dynamics of neurogenesis in a brain area relevant to song control. Given that effects were bilateral, while T or E2 implants were unilateral, these neuroanatomical effects are likely to be activity-dependent although the numbers of multipolar DCX-ir cells in HVC did not correlate with the measures of song that were affected by the treatments, namely the number of songs and the percentage of time spent singing (all *p* ≥ 0.141). Interestingly also, the effect was limited to HVC and not seen in two adjacent areas thus stressing again that, as observed before (Balthazart et al., 2008), neurogenesis and recruitment of new neurons is controlled in a specific manner within this song control nucleus.

Interestingly, although E2 implanted in POM produced nearly identical effects on vocal behavior, this treatment did not affect DCX-ir multipolar cells in HVC. This differential effect of T and E2 might reflect a differential time course of action so that the new neurons would have been sampled at a different latency after their final mitotic division in T and E2 birds. This difference affecting DCX-ir cells may indeed relate to the fact already discussed before that the maximal effects of T on song were observed on day 14 but only on day 28 in E2 birds. All these data clearly point to the fact that we would need more studies on the time course of neurogenesis in HVC.

### PNNs and PV-ir neurons

Although PNN density and/or total numbers in HVC are increased by systemic T treatment in adult male canaries (Cornez et al., 2017a), no change was detected here after implantation of T or E2 in the POM. The increase of PNN density in HVC has been hypothesized to play a key role in song crystallization of song by stabilizing synaptic connections of specific subsets of neurons (Balmer et al., 2009; Cornez et al., 2017b, 2018b). However, no study to date has attempted to determine whether this increase in PNN density is due to a direct effect of T on HVC or is, like neurogenesis, driven at least in part by the singing activity itself. Females bearing a T or E2 implant in POM displayed here an increase in vocalizations, but no change in PNN expression. This observation might be construed to conclude that the PNN expression is not activity dependent, but is rather controlled by a direct action of steroids in HVC. A major limitation to this conclusion is, however, that the vocal activity induced here by steroids was quite limited both in quantity and quality. The songs produced by these females also never showed the features of crystalized song so that it makes sense that PNN expression was not increased and actually remained at a very low level comparable to what is observed in females not treated with T (Cornez et al., 2017a) and much below what is seen in sexually mature males (Cornez et al., 2018a) or castrated males treated with exogenous T (Cornez et al., 2017a). Additional studies independently manipulating direct action of T in HVC and singing activity would be needed to reach formal conclusions on this question.

In conclusion, the present study indicates that as observed in males, sex steroids increase the motivation to sing in female canaries by acting in the medial preoptic area and they correlatively increase neurogenesis in HVC. However, as observed after systemic treatments with T, female songs do not reach the same level of quality and are not produced as frequently as male songs. Future research should investigate whether longer treatments or treatments with higher doses of T might be able to overcome this sex difference or if it relates to organizational effects of early exposure to sex steroids or even to direct genetic effects independent of gonadal steroid hormone action.

## Synthesis

Reviewing Editor: Rae Silver, Columbia University Decisions are customarily a result of the Reviewing Editor and the peer reviewers coming together and discussing their recommendations until a consensus is reached. When revisions are invited, a fact-based synthesis statement explaining their decision and outlining what is needed to prepare a revision will be listed below. The following reviewer(s) agreed to reveal their identity: Stephen Nowicki.

The manuscript describes a study demonstrating that both T and E2 applied to the medial preoptic area induces female canaries to increase their production of male-like songs. Specifically, local, unilateral administration of T or E2 directly into the POM of the adult female canary can increase singing and produce measurable anatomical responses. Both of these results seem reasonably well-supported. The authors demonstrate that T increases song more rapidly post-administration than E2, by comparing birds in which the steroids were delivered into the POM, with birds in which the very same steroids missed the POM. Correlated with this, there is an apparent increase in neurogenesis (as reflected by the number of a subset of DBX-ir neurons) in HVC in females that received steroids into the POM relative to those in which the steroids missed the POM. There was no effect of the location of steroid administration on other neuroanatomical or neurochemical measures. Implants did not apparently affect circulating levels of T as measured by cloacal protuberance and syrinx weight.

The study did not include a control group per se, but rather used individuals for which post mortem histology revealed that the implanted androgen missed the POM target as a kind of negative control. I think this approach is reasonable, given that a significant percentage of misses was expected. However, this point needs to be explained more clearly and much sooner in the manuscript, preferably in the final paragraph of the Introduction.

Relatively small sample sizes represent the most significant weakness of the manuscript because of the difficulty in interpreting negative results. A number of comparisons are found to be non-significant, but it's difficult to know if this is simply the result of sample sizes that are too small to detect differences.

The statistics brought to bear throughout the paper need to be more clearly explained, including the sample sizes involved, with the latter being sometimes hard to follow given that individuals are dropped from particular comparisons for a variety of reasons (such lack of sufficient song recordings, accidental loss of data, etc.). In particular, I was left wondering why a combination of repeated measures ANOVAs and mixed effects models was used. Given that mixed effects models are used for some analyses, why not all of them? Also, performing multiple ANOVAs, if I understand would require a correction for multiple comparisons (e.g., a Bonferroni adjustment of significance levels). Mixed effects models would be a preferred way to go anyway, and would likely make it easier to explain statistical results more clearly overall.

With respect to sample sizes: why were all 16 birds in the first cohort were given T, while in the second cohort 4 birds were given T and 12 given E2. What was the rationale behind this decision? Related to this: The authors justify combining of T and E2 administration outside the POM into a single experimental group. This is described as a series of statistical comparisons only a small subset of which are actually presented as text in the manuscript (effect on singing). For the remainder, the authors simply state that, “..none of the 26 t-tests.....,” except for one revealed a significant difference. I'm sorry if I've missed something, but the rest of the text simply presents the combined data (text and figures) - thereby preventing the reader from evaluating the means and std errors of T outside POM compared to E2 outside POM. The entire paper hinges on this comparison. The authors are strongly urged to present the individual data points of the OUT group in all the figures (perhaps as points on the bar graph) and clearly demarcate which ones were T and E2, thereby permitting the reader a better understand of the variance in this experimental group. Since this is a group that is kluged post-hoc, it is important that the reader sees at a glance that there truly was no differences between T and E2 outside POM. Alternatively, the authors may want to present a table that describes the means, errors, and statistical comparisons of all 26 t-tests. Either scenario would permit a more thorough, and therefore better understanding of the experiment and results.

The description of the song analysis and rationale for why particular parameters were measured also need to be spelled out more completely. The amount of singing and the duration of songs produced both make sense, as does a measure of bandwidth. Entropy (as measured by Raven Pro) also makes sense, although here what this parameter actually measures needs to be spelled out and it will be more useful to compare values to values obtained for typical male song. What's less clear is why power and three measures of amplitude are all included in the analysis. Is there a prediction that more male-like songs are higher amplitude? Certainly, the three amplitude measures are likely to be highly correlated, so why measure all three? Finally, was acoustic “power” actually measured? To calculate power, one needs to make recordings with a calibrated measuring microphone positioned at a fixed distance and angle from the bird, but I don't see any indication this was done. The manuscript refers to song “complexity” in a couple of places (lines 433 & 440), but none of the song features that were quantified address complexity in a meaningful way. Finally, the results refer to the high variability of female song and state that this level of variation is similar to male plastic song (line 443 ff), but this point is not supported by any data presented (or even a published reference to male plastic song). This claim should either be better supported or deleted.

Figure 2 nicely schematizes the location of the tips of the needles used for delivery. It would be more informative and interesting to see this figure supplemented with a photomicrograph of the delivery location either in Nissl or immunostained tissue. This would permit a better understanding of the size of the needle tract and the actual injection site. The POM is a small nucleus and the extent of damage to the nucleus during a pressure injection of crystalline steroid is worthy of evaluation. While the aromatase and DBX icc clearly shows apparently healthy neural tissue, I am left with a question as to how far the administered steroid diffused and how much tissue was affected by the delivery.

Minor concerns

There are a number of places where the vocabulary used appears strange. Eg. “focalized,” rather than focused. Please review carefully and correct as necessary.
